# Investigating the Effects of Auditory and Vibrotactile Rhythmic Sensory Stimulation on Depression: An EEG Pilot Study

**DOI:** 10.7759/cureus.22557

**Published:** 2022-02-24

**Authors:** Abdullah A Mosabbir, Thenile Braun Janzen, Maryam Al Shirawi, Susan Rotzinger, Sidney H Kennedy, Faranak Farzan, Jed Meltzer, Lee Bartel

**Affiliations:** 1 Rotman Research Institute, Baycrest Health Sciences, Toronto, CAN; 2 Faculty of Music, University of Toronto, Toronto, CAN; 3 Department of Psychiatry, University Health Network, Toronto, CAN; 4 Centre for Depression and Suicide Studies, St. Michael’s Hospital, Toronto, CAN; 5 School of Mechatronic Systems Engineering, Simon Fraser University, Surrey, CAN

**Keywords:** vibrotactile, vibroacoustic stimulation, gamma stimulation, rhythmic sensory stimulation, electroencephalography, depression

## Abstract

Background

Major depressive disorder (MDD) is a persistent psychiatric condition and one of the leading causes of global disease burden. In a previous study, we investigated the effects of a five-week intervention consisting of rhythmic gamma frequency (30-70 Hz) vibroacoustic stimulation in 20 patients formally diagnosed with MDD. In that study, the findings suggested a significant clinical improvement in depression symptoms as measured using the Montgomery-Asberg Depression Rating Scale (MADRS), with 37% of participants meeting the criteria for clinical response. The goal of the present research was to examine possible changes from baseline to posttreatment in resting-state electroencephalography (EEG) recordings using the same treatment protocol and to characterize basic changes in EEG related to treatment response.

Materials and methods

The study sample consisted of 19 individuals aged 18-70 years with a clinical diagnosis of MDD. The participants were assessed before and after a five-week treatment period, which consisted of listening to an instrumental musical track on a vibroacoustic device, delivering auditory and vibrotactile stimulus in the gamma-band range (30-70 Hz, with particular emphasis on 40 Hz). The primary outcome measure was the change in Montgomery-Asberg Depression Rating Scale (MADRS) from baseline to posttreatment and resting-state EEG.

Results

Analysis comparing MADRS score at baseline and post-intervention indicated a significant change in the severity of depression symptoms after five weeks (t = 3.9923, df = 18, p = 0.0009). The clinical response rate was 36.85%. Resting-state EEG power analysis revealed a significant increase in occipital alpha power (t = -2.149, df = 18, p = 0.04548), as well as an increase in the prefrontal gamma power of the responders (t = 2.8079, df = 13.431, p = 0.01442).

Conclusions

The results indicate that improvements in MADRS scores after rhythmic sensory stimulation (RSS) were accompanied by an increase in alpha power in the occipital region and an increase in gamma in the prefrontal region, thus suggesting treatment effects on cortical activity in depression. The results of this pilot study will help inform subsequent controlled studies evaluating whether treatment response to vibroacoustic stimulation constitutes a real and replicable reduction of depressive symptoms and to characterize the underlying mechanisms.

## Introduction

Major depressive disorder (MDD) is a highly prevalent and persistent psychiatric condition that is regarded as one of the leading causes of global disease burden [1,2]. MDD is broadly characterized by at least one of two core symptoms - persistent depressed mood and/or diminished interest or pleasure - accompanied by other identified psychological symptoms present for at least two weeks [1,2]. Although antidepressant medication is the first line of treatment for MDD, a sizeable percentage of patients do not respond to medication even after several treatment attempts as the effects of the many available antidepressants are inconsistent [3]. This unmeet need for treatments with optimal clinical response and rapid onset of benefit has led to the investigation of brain-based biomarkers to predict the likelihood of a patient benefiting from a certain medication or therapeutic approach, thus optimizing treatment selection and assisting in the development of new treatment alternatives [4-8].

Biomarkers are objective measures of pharmacological response or biological processes that are quantifiable, precise, and reproducible [9]. A promising neurophysiological biomarker is an electroencephalography (EEG). Reports on putative EEG-related biomarkers as predictive of treatment response have been ongoing for many decades, with several comprehensive reviews on this topic [10-12]. The common EEG biomarkers used for predicting response to treatment (at baseline) or as indicative of response (posttreatment versus pretreatment) include measures of change in the activity of EEG canonical frequency bands, hemispheric alpha asymmetry [13-15], theta cordance [16-18], or the antidepressant treatment response index [17,18].

Changes in oscillatory brain activity are also useful for diagnostic purposes and provide relevant information regarding the mechanisms underlying the disorder [19-22]. Of particular interest in the present study are alterations in gamma-band activity in unipolar depression [23,24]. Gamma oscillations are relatively high-frequency (>30 Hz) components of the EEG and have been associated with sensory and cognitive functions and neural plasticity [25]. Recently emerging neurophysiological evidence suggests that alterations in gamma-band oscillations in individuals with unipolar depression compared to healthy controls are associated with mood swings [23], negative response bias during emotional face processing [26], and higher cognitive reactivity in a lexical emotion identification task [27]. Reduced resting gamma at baseline was also found in subjects with elevated depression symptoms [28]. Importantly, it has been consistently shown that pharmacological and non-pharmacological treatments that counteract depression symptoms induce changes in gamma activity, suggesting that gamma oscillations may also be markers of treatment recovery or mediators of therapeutic effect [23,29,30].

The clinical potential of gamma-band modulation with rhythmic sensory stimulation (RSS) has received increased attention in recent years [31,32]. This is in part due to a series of studies conducted in mouse models of Alzheimer’s disease where it was demonstrated that the delivery of gamma (i.e., 40 Hz) auditory-visual stimulation significantly improved multiple dementia-related biomarkers by inducing neuroprotective mechanisms in several brain areas [33-35]. One of the hypotheses for the underlying mechanism is that repetitive gamma sensory stimulation would entrain local brain oscillatory activity at gamma frequencies [36,37]. To date, evidence of the therapeutic effects of gamma sensory stimulation is at its initial stages [38-41]. In a previous open-label study [42], we explored the potential effects of sound-driven vibrotactile stimulation on depression. Gamma frequency (30-70 Hz) RSS was embedded in designed instrumental music and delivered for a total of five weeks using a portable consumer device with built-in stereo speakers and a low-frequency transducer, generating both auditory and vibrotactile stimulations. The results indicated a significant improvement from baseline in depressive symptoms and benefits in associated symptoms including sleep quality, quality of life, anhedonia, and music-reward processing.

In the present study, we further investigated the effects of RSS on depression. The current paper aims to examine whether the intervention induces changes from baseline to posttreatment in resting-state EEG recordings and to report basic changes in EEG between responders and nonresponders in the same cohort of depressed participants from our previous study. The analysis focused on basic oscillatory power measurements in canonical frequency bands. We hypothesized that there would be modulation in resting gamma-band frequency posttreatment compared to pretreatment and that responders would differ from nonresponders in gamma activity after the intervention. Given that there have been mixed results in studies investigating the predictive utility of EEG frequency bands, we did not have specific hypotheses regarding other EEG biomarkers.

## Materials and methods

Study design and ethics statement

This is an open-label pilot study containing a single group of patients diagnosed with major depressive disorder (MDD). The study is a collaboration between the Faculty of Music at the University of Toronto, the Canadian Biomarker Integration Network in Depression (CAN-BIND), and the Baycrest Health Sciences Center. The protocol was approved by the University Health Network Research Ethics Board (15-9799-AE) and registered at ClinicalTrials.Gov (NCT02685982). Written informed consent was obtained from all participants in accordance with the Declaration of Helsinki.

Participants

The study sample consisted of 20 individuals aged 18-70 years with a clinical diagnosis of MDD and currently experiencing a major depressive episode (MDE). The severity of illness was evaluated using the Montgomery-Asberg Depression Rating Scale (MADRS), and the participants were required to score ≥15 [43]. Participants were excluded if they had any Axis I diagnosis (other than MDD) that was considered the primary diagnosis, MDD with psychotic features, a diagnosis of bipolar disorder type I or II, a significant Axis II diagnosis (borderline and antisocial), a formal diagnosis of fibromyalgia, high suicidal risk, substance dependence/abuse in the past six months, and presence of a significant neurological disorder, head trauma or other unstable medical condition. Other reasons for exclusion included any change in medication type or dosage four weeks prior to enrollment or beginning psychological treatment up to three months prior to enrollment. Female participants who were pregnant or breastfeeding were also excluded. A total of 23 participants were screened, of whom three were excluded due to a recent change in medication dosage or substance misuse. Twenty participants were enrolled and provided informed consent, although one withdrew and did not complete the posttreatment outcome measures. The remaining 19 participants were included in the analyses. The participants received a gift certificate for $100 CAD.

Intervention

The intervention consisted of listening to an instrumental musical track embedded with low-pitch sounds on the gamma-band range (30-70 Hz, with particular emphasis on 40 Hz) and binaural detunement at 10-15 Hz whereby different auditory stimuli were presented simultaneously to each ear. The intervention was delivered using a portable consumer device (Sound Oasis Vibroacoustic Therapy System VTS-1000, Sound Oasis, Marblehead, MA, USA; “Energize” soundtrack) with built-in stereo speakers and a low-frequency transducer, which allowed for the low-pitch sounds embedded in the music to be experienced as a mild vibrotactile sensation around the lower-back area of the torso and the presentation of the auditory effects embedded in the music. The intervention was self-administered at home for 30 minutes, five days per week, over five weeks. The participants were instructed to place the device on a chair/bed and relax for the duration of the session, with no specific restrictions on the activities that could be performed during the session. Treatment logs were used to confirm the number of sessions completed, the type of activities performed during each session, and the device settings for the volume of the music and the intensity of the vibrotactile stimulation. Study compliance was assessed via phone or e-mail communication at weeks 2 and 4 of the intervention, as well as through the treatment logs submitted at the final visit.

Clinical measures

The participants were assessed before and after the five-week treatment. The primary clinical endpoint was the change in MADRS from baseline to posttreatment. This clinician-based measure consists of 10 items rated on a 6-point scale, with 0 being “normal/not present” and 6 being “extreme.” MADRS total scores range from 0 to 60, with higher scores indicating more severe symptoms. “Responders” were defined as those who achieved ≥50% decrease in MADRS scores from baseline to endpoint, while those with <50% decrease were defined as “nonresponders.”

EEG recording

Resting-state EEG was recorded for each subject at baseline and posttreatment visits and consisted of two eight-minute periods containing eyes-open and eyes-closed conditions. During the eyes-open condition, the participants were instructed to fix their gaze centrally on a computer screen and to remain still to minimize any movements or eye blinks.

Recordings were performed using a BioSemi Active Two amplifier system (BioSemi, Amsterdam, The Netherlands) with 64 channels, using active Ag/AgCl electrodes mounted on an elastic cap. Eight additional electrodes were placed below the hairline (both mastoids, both preauricular points, outer canthus of each eye, and inferior orbit of each eye). Eye movements were recorded with the electrodes placed at the outer canthi (horizontal electrooculogram (EOG)) and at the inferior orbits as vertical EOG. Two further electrodes (Common Mode Sense (CMS) active electrode and Driven Right Leg (DRL) passive electrode) were used as reference and ground electrodes, respectively (cf. www.biosemi/faq/cms&drl.htm). Data were collected with a sampling rate of 512 Hz with a low-pass cutoff of 102.4 Hz.

EEG data analysis

The EEG recording system, data pre-processing, and analysis followed the standardized procedure of the EEG working group of the Canadian Biomarker Integration Network in Depression (CAN-BIND) [5,44]. Data pre-processing was performed using EEGLAB, an open-source, MATLAB-based suite for EEG data processing. All data were subjected to a bandpass filter of 1-80 Hz and a bandstop filter of 55-65 Hz and subsequently segmented into 1s epochs. Epochs and channels with noise were excluded from analyses by visual inspection. Independent component analysis (ICA) was then performed to remove artifacts, such as eye blinks, eye movements, and muscle artifacts. This was followed by a second round of visual inspection for noisy channels and epochs. Noisy channels were interpolated, and recordings were re-referenced to the average reference. The EEG data were then subjected to a power spectrum analysis using Welch’s method. Absolute power (μV2) was calculated for each of the five frequency bands: delta (1-3.5 Hz), theta (4-7 Hz), alpha (8-12 Hz), beta (12-30 Hz), and gamma (30-60 Hz). Following the procedures from Baskaran et. al, in order to reduce the amount of data in summary statistical analyses of frequency band power, four medial electrode sites subdivided by hemisphere were chosen: frontal (left/right: F3/4), central (C3/4), parietal (P3/4), and occipital (O1/2) regions [5].

Statistical analysis

For the clinical outcome measure, MADRS scores pre- and post-intervention were compared using paired t-tests. Statistical analyses of EEG measures focused on frequency band power at pre- and post-intervention. Absolute frequency band power was analyzed using a mixed-model repeated measures analysis of covariance (ANCOVA) with the percent of change in MADRS score as a covariate and the following within-subject factors: timepoint (before and after) and region (prefrontal, frontal, central, parietal, and occipital). Post hoc analyses between groups were performed using dependent or independent sample t-tests as appropriate. Pearson’s correlations between changes in EEG power and changes in MADRS scores were performed for all EEG parameters that identified significant pre-changes versus post-changes across the entire group. All analyses were carried out using the R software.

## Results

Clinical measures

**Figure 1:**
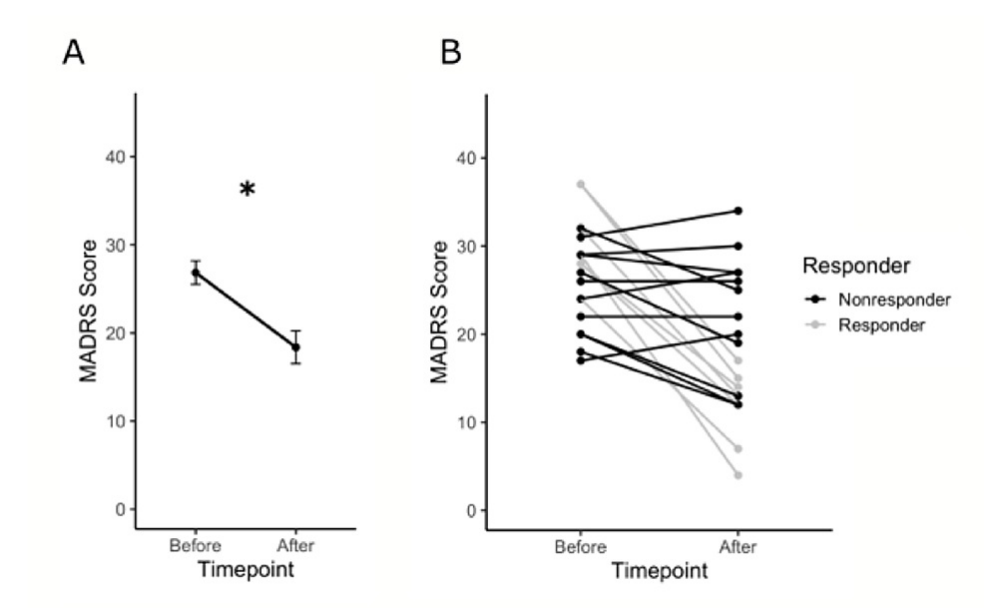
Participant MADRS scores. A) The change in MADRS scores before and after RSS treatment for the entire group. Asterisk depicts a significant difference (p = 0.0008). B) The change in MADRS scores for each individual before and after RSS treatment. Responders and nonresponders are colored gray and black, respectively, and response to treatment is defined by an improvement in MADRS of 50%.

Analysis comparing MADRS scores at baseline and post-intervention indicated a significant change in the severity of depression symptoms after five weeks (t = 3.9923, df = 18, p = 0.0009) (Figure 1A). The clinical response rate was 36.85% (n = 7), and the nonresponse rate was 63.15% (n = 12) (Figure 1B). A summary of the demographic and clinical characteristics of the study sample is presented in Table 1.

**Table 1:** Demographic and clinical characteristics of the study participants. Values are expressed as mean ± standard deviation or count.

	Responders (n = 7)	Nonresponders (n = 12)	Total (n = 19)
Age (years)	49.5 ± 9.3	46.8 ± 12.6	47.8 ± 11.3
Sex (female/male)	5/2	6/6	11/8
Marital status			
Never married	0	6	6
Married/partnered	2	5	7
Divorced/separated	5	1	6
Education			
High school	2	4	6
College/no degree	2	1	3
College/university degree	3	7	10
Occupational status			
Full-time employed	2	1	3
Unemployed, looking for work	4	2	6
Student	0	2	2
Keeping house	0	1	1
Disabled	0	5	5
Retired	1	1	2
Psychiatric medication (yes/no)	5/2	8/4	13/6
Baseline MADRS score (0–60)	30.71 ± 4.88	24.58 ± 5.16	26.84 ± 5.78
Post-intervention MADRS score (0–60)	11.71 ± 4.66	22.25 ± 7.23	18.36 ± 8.14

Frequency band power after treatment

The analysis of absolute frequency band power in the eyes-closed condition did not show any significant difference pretreatment versus posttreatment for any frequency band across the entire group. The alpha power in the eyes-closed condition was significantly greater than the eyes-open condition in the occipital regions for the entire group pre- and posttreatment, as expected (data not shown). In the eyes-open condition, there were no significant changes in the delta, theta, or beta frequency bands. Significant differences in power pretreatment versus posttreatment in the eyes-open condition were found for alpha and gamma. Relative power did not show notable results to a significant degree.

**Figure 2:**
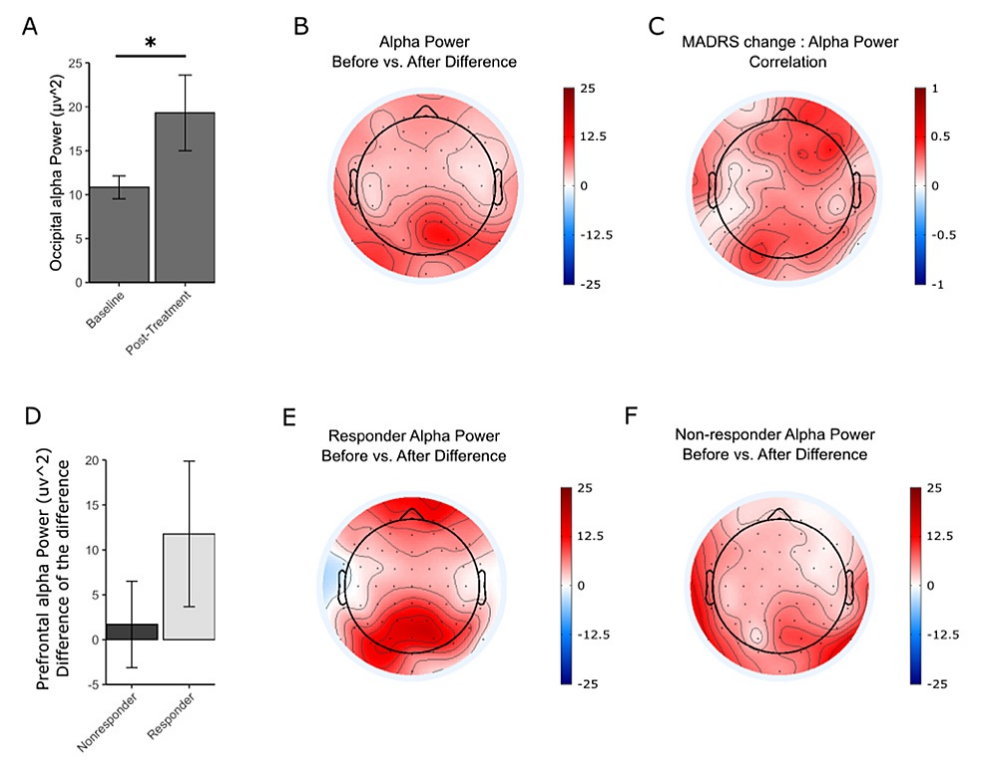
Resting-state alpha power before and after treatment. A) Alpha power in the occipital electrodes. Asterisk depicts a significant difference (p = 0.045). B) Topographical map of the change in alpha power for the entire group across all electrode regions. C) Topographical map showing the correlation coefficient R between the change in alpha power and the change in MADRS at each electrode. D) The change from baseline to posttreatment in prefrontal alpha power (t = 1.0692, df = 10.269, p = 0.3095). E) Topographic map of the change in alpha for responders across electrodes. F) Topographic maps of the change in alpha for nonresponders.

Alpha power increased for patients with MDD after the treatment period. The increase in alpha power was greatest in the occipital area (Figure 2A-2B). The ANCOVA results indicate a significant interaction of percent of change in MADRS and timepoint (F(1,275) = 21.11, p = 6.67 × 10-6). Post hoc results showed a significant increase in occipital alpha power (t = -2.149, df = 18, p = 0.04548) (Figure 2A). A regression was performed to measure the correlation between the MADRS change and the alpha power change in each electrode, and the results are plotted as a topographic map showing greater values in the frontal and occipital regions (Figure 2C). Changes in the alpha power between responders and nonresponders were also explored. Although the responders showed a greater increase in the prefrontal and occipital regions, this was not significantly different from the nonresponders (Figure 2D-2F). 

**Figure 3:**
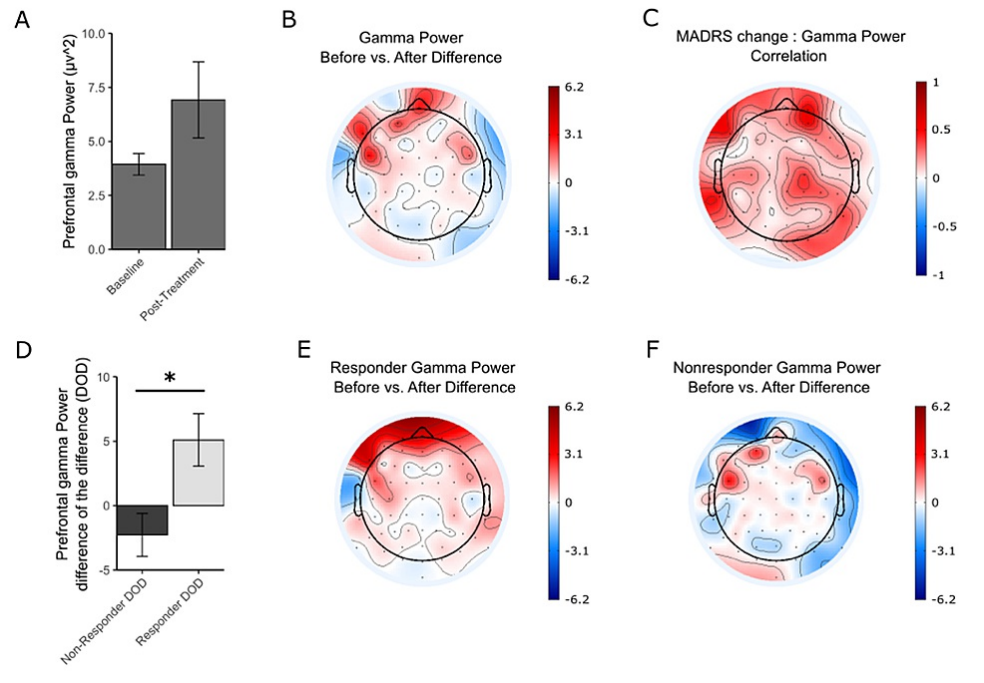
Resting-state gamma power before and after the treatment. A) Gamma power at the prefrontal electrodes (p = 0.1135). B) Topographical map of the change in gamma power for the entire group. C) Topographical map showing the correlation between the change in gamma power and the change in MADRS at each electrode. D) Gamma power of responders and nonresponders before and after treatment. DOD refers to the difference of gamma power before versus after treatment of the nonresponders and responders. Asterisk indicates a significant difference (p = 0.01442). E) Topographical map of the change in gamma power for the responder group. F) Topographical map of the change in gamma power for the nonresponder group.

Gamma power also increased for patients with MDD after the treatment period. The increase in gamma power was greatest in the prefrontal area (Figure 3A-3B). The ANCOVA results indicate a significant three-way interaction of percent change in MADRS, timepoint, and region (F(4,275) = 3.25, p = 0.013). There was also an interaction of MADRS and timepoint (F(1,275) = 5.01, p =0.025), as well as a main effect of region (F(4,68) = 7.68, p = 3.615 × 10-5). A post hoc comparison focusing on the prefrontal electrodes Fp1 and Fp2 (included in the ANCOVA model) showed a nonsignificant increase in prefrontal gamma power across all participants (t = -1.6637, df = 18, p = 0.1135) (Figure 3A). A regression was done to measure the correlation between the MADRS change and the gamma power change in each electrode and plotted as a topographic map (Figure 3C). Changes in gamma power between responders and nonresponders were also explored. There was a significant increase in gamma power in the prefrontal regions for responders, but not for the nonresponders (5.10 ± 3.1 for responders and -2.276 ± 1.85 for nonresponders). The change in power posttreatment versus pretreatment was significantly larger in responders versus nonresponders (t = 2.8079, df = 13.431, p = 0.01442) (Figure 3D). Topographic maps support these findings and show the prefrontal area as the main region of difference before and after treatment for responders, but not for nonresponders (Figure 3E-3F).

## Discussion

This study examined EEG-derived biomarkers to evaluate the effects of sound-driven RSS on depression. We examined changes in resting-state EEG pre- to posttreatment and characterized basic changes in EEG in relation to treatment response. Our results indicate that improvements in depressive symptoms after five weeks of RSS were accompanied by an increase in alpha power in the occipital region and an increase in gamma in the prefrontal region after the treatment period.

A prominent finding in this study was an increase in alpha power in the occipital electrodes after the treatment period. Pretreatment differences in alpha-band activity have been consistently found in pharmacological studies, with responders tending to have greater alpha power than nonresponders at baseline [14,45,46]. However, studies focusing on pretreatment versus posttreatment changes indicate that alpha power either does not change or it decreases with some antidepressant treatments [14,47-49]. Therefore, it is possible that the modulation of neural oscillations induced by RSS may not relate to the same mechanisms.

RSS is a form of pulsed stimulation via sensory input pathways, while other forms of stimulation using transcranial magnetic stimulation (TMS) modulate brain activity bypassing sensory input [50]. Increased alpha oscillatory activity restricted to the area of stimulation is a typical response to repetitive TMS (rTMS) at various frequencies (e.g., single pulse, 1 Hz, 10 Hz, and 20 Hz) [51-53]. Studies investigating rTMS on MDD patients typically stimulate alpha frequency rTMS at the dorsolateral prefrontal cortex [54-56]. In several studies, patients with MDD stimulated in this region showed an increase in frontal alpha power, which has been associated with significant improvements in clinical symptoms [54-56]. Interestingly, one study demonstrated that the rTMS stimulation of the medial prefrontal cortex (which is part of the default mode network (DMN)) could induce an increase in occipital alpha power that lasted even after the discontinuation of the stimulation [52]. This study argued for a strong coupling of the DMN and occipital alpha power, suggesting that the stimulation of this network can induce such an effect. Therefore, it is possible that the increased occipital alpha activity seen in the current study may be related to a long-lasting effect of pulsed stimulation compounded over five weeks of RSS.

Another notable effect of RSS was an increase in prefrontal gamma power that was greater in the responders than in the nonresponders. The use of gamma activity as a biomarker of treatment response in depression is an emerging topic [23,57,58]. It has been shown that different classes of antidepressant drugs have distinct effects on gamma oscillations, whereby serotonin-boosting antidepressants (e.g., citalopram and fluoxetine) suppress gamma, while noradrenergic drugs and ketamine increase gamma activity [59-64]. These opposing effects suggest that there may be different mechanisms of action that may be associated with distinct treatment responses. This may potentially explain why RSS was effective only for some patients with MDD. Ketamine is an effective treatment for depression, and the most well-characterized and prominent effect of ketamine on resting EEG is an increase in gamma power, although this is not necessarily associated with a reduction in depressive symptoms. Studies on the effects of ketamine on depression have demonstrated increased gamma power in the frontal and prefrontal area, with effects lasting up to nine hours after medication intake [23,63,65-68]. Non-pharmacological treatments for depression have also induced increases in gamma signaling, particularly in the resting state, associated with symptomatic improvement [29,69,70]. Pulsed stimulation with rTMS has shown an increase in gamma activity in the prefrontal area [29]. Collectively, these studies suggest that a long-lasting increase in gamma activity may be an indicator of treatment response for MDD.

Changes in EEG power may also have been impacted by merely listening to music, as there is a musical component to the intervention used in this study. It has been shown that auditory rhythms consisting of pure tones entrain endogenous activity that corresponds to the beat of the music, especially in delta and theta frequency bands [71-77]. Listening to music also engages neural activity across multiple frequency bands that are associated with the perception and processing of music features during naturalistic music listening [78-82]. Short-term music listening can elicit an increase in alpha power usually localized in frontal and temporal regions, whereas an increase in posterior alpha is usually associated with imagining music, which likely did not occur in this study [83]. With regard to resting-state gamma power, it has been shown that music listening induces an overall decrease in gamma, especially in the prefrontal area [84]. While these findings refer to the immediate effects of music on the entrainment of brain oscillations, the long-term effects of music listening on EEG power spectra have not yet been clearly described. It is important to note, however, that the music presented in this intervention was designed to emphasize gamma-band frequencies and it has been well established that the entrainment is maximal at specific frequencies rather than a mix of different frequencies [50]. Moreover, we hypothesize that the effects observed in the present study are related to a long-lasting effect of pulsed stimulation compounded over five weeks of RSS; thus, any supposed neural entrainment induced by RSS cannot be definitively associated with its “musical” nature more so than the pulsed nature of RSS. Further controlled studies are needed to better determine the role of music listening and rhythmic sensory stimulation on the effects induced on depression in this intervention.

Limitations

A major limitation of this study, however, is that as a pilot study, there was no control group nor a placebo group, so the EEG measurements cannot be differentiated from the placebo effect. However, the results of this study encourage larger, randomized, placebo-controlled studies.

## Conclusions

In conclusion, music and vibroacoustic-based RSS had a positive and significant effect on improving the depressive symptoms of patients with MDD, with a clinical response rate of 37%. These changes were accompanied by an increase in alpha power in the occipital region and an increase in gamma in the prefrontal region, suggesting treatment effects on cortical activity in depression. The EEG biomarkers observed after RSS treatment replicated the findings of other well-known MDD treatments, including rTMS and ketamine.

As this pilot study did not include a control group, there is also a possibility of placebo effects contributing to the observed changes, both in clinical outcomes and EEG measures. Thus, the result of this study encourages future placebo-controlled studies with RSS. Nevertheless, even if the reduction in depressive symptoms in the present study were to be attributable solely to placebo effects, it is still of interest to characterize the neurophysiological changes associated with such a response, so that the physiological mechanisms underlying responses to drugs and other noninvasive stimulation techniques (e.g., TMS) can be compared. Future testing could also examine how single frequency bands interact with other EEG biomarkers of depression including other frequency bands, measurements such as cordance, or the antidepressant treatment response index. Research investigating the fundamental components of RSS (e.g., music, frequency-specific sound, and vibrotactile stimulation) in relation to clinical symptom improvements is also needed. The results of this pilot study will help inform subsequent controlled studies evaluating whether treatment response to vibroacoustic stimulation constitutes a real and replicable reduction of depressive symptoms and characterize the underlying mechanisms.
